# Availability, pricing and affordability of essential medicines in Eastern Ethiopia: a comprehensive analysis using WHO/HAI methodology

**DOI:** 10.1186/s40545-021-00339-2

**Published:** 2021-07-05

**Authors:** Mekonnen Sisay, Firehiwot Amare, Bisrat Hagos, Dumessa Edessa

**Affiliations:** 1 Department of Pharmacology and Toxicology, School of Pharmacy, College of Health and Medical Sciences, Haramaya University, P.O. Box 235, Harar, Ethiopia; 2 Department of Clinical Pharmacy, School of Pharmacy, College of Health and Medical Sciences, Haramaya University, P.O. Box 235, Harar, Ethiopia; 3 Department of Social Pharmacy, School of Pharmacy, College of Health and Medical Sciences, Haramaya University, P.O. Box 235, Harar, Ethiopia

**Keywords:** Availability, Price, Affordability, Essential medicines, Medicine outlets, WHO/HAI

## Abstract

**Background:**

Access to essential medicines is a universal human right and availability and affordability are the preconditions for it. In line with the sustainable development goals, World Health Organization (WHO) has outlined a framework that assists the policy makers to improve access to essential medicines for universal health coverage by 2030. However, the availability and affordability of essential medicines remains suboptimal in several low-income countries. Therefore, this study was designed to investigate the availability, pricing and affordability of essential medicines in eastern Ethiopia.

**Methods:**

A cross-sectional study design was employed to conduct this study. Public and private health facilities found in Eastern Ethiopia and which fulfilled criteria set forth by WHO/Health Action International (HAI) guideline and essential medicines listed on WHO/HAI guideline and essential medicine list of Ethiopia were included. Accordingly, 60 medicine outlets were selected based on the WHO/HAI standardized sampling methodology. A standardized data collection tools developed by WHO/HAI, with necessary modifications, was employed to collect the data. Median Price Ratio (MPR) was computed as a ratio of median local buyers’ price to international buyers’ reference price. The Mann–Whitney *U* test was employed to compare the median buyers’ price between public and private health facilities. Kruskal–Wallis test was also run to explore the median price difference among all facilities. Treatment affordability was calculated based on the number of days of wage of the lowest-paid government employee of Ethiopia required to purchase the prescribed regimen.

**Results:**

The overall percent availability of originator brand (OB) versions of essential medicines was found to be 3.6% (range: 0.0–31.7%), with the public and private sectors contributing 1.43% and 5.50%, respectively. The overall percent availability of lowest price generics (LPGs) was 46.97% (range: 1.7–93.3%) (Public: 42.5%; private: 50.8%). Only eight LPGs (16.0%) met the WHO target of 80%. The Mann–Whitney *U* test indicated that 64% drugs showed statistically significant median price difference between public and private settings (*p* < 0.05). The MPR value indicated that the median buyers’ price of drugs in private sector were more than four times the international reference price in 30% of drugs. The percentage of unaffordable medicine were 72.09 and 91.84% for public and private facilities, respectively, with 79.17% of the medicines were unaffordable when both settings were combined.

**Conclusion:**

Only 16% of the surveyed medicines surpassed the WHO cut-off point of 80%. Nearly one-third of drugs in the private sector had a price of more than four times compared to the international reference prices. Moreover, four out of five drugs were found unaffordable when both settings were combined, demanded several days of wage of lowest paid government employee. This finding calls a prompt action from stakeholders to devise a strategy that help promote the access of essential medicines and rescue the struggling healthcare system of Ethiopia.

## Background

Health is a fundamental human right while access to health care is a way of ensuring the fulfillment of this right [1]. Universal health coverage (UHC) as the main target of sustainable development goal strives to achieve access to quality health services according to the need, while ensuring imposition of less financial hardship on the users of the services [2]. The realization of UHC have the access of essential medicines (EMs) at its core as they are an indispensable element for delivery of services and a requirement for high-quality care [3].

Availability and affordability are dimensions of measures of access to medicines in health systems [4]. Access to affordable, quality-assured EMs is crucial to reducing the financial burden of care, preventing greater pain and suffering, shortening the duration of illness, and averting needless disabilities and deaths worldwide [5]. However, one third of the world’s population lacks regular access to EMs, resulting a cascade of preventable misery and suffering [6, 7]. This estimate rises to over 40% in low-income countries and over 50% in poorest countries of Asia and Africa despite the issuance of legislations supporting the implementation of UHC by the countries [8].

EMs satisfy the priority health care needs of the population. They are intended to be available within the context of functioning health systems at all times in adequate amounts, in the appropriate dosage forms, with assured quality, and at a price, the individual and the community can afford [9, 10]. In many developing countries, lack of financial resources or information can create barriers to accessing essential medicines and contributing for the increased rate of morbidity and mortality [11, 12]. On top of unavailability of EMs, high price of medicines is posing a problem in the provision of health services [13]. Price of medicines is a concern in low- and middle-income countries where up to 90% of the population purchases medicines through out-of-pocket payments [14]. This has a huge impact on the affordability of medicines and treatment outcome of diseases [15].

In the mid-1990s, civil society organizations in developed and developing countries started drawing attention to the need for increased access to essential medicines as part of the fight against poverty. Later, the World Health Organization/Health Action International (WHO/HAI) project was established in 2001 to deal with medicine prices and availability. The main focus of this project was to develop a reliable methodology for collecting and analyzing availability, affordability and medicine price component data across healthcare sectors and regions; to publish survey data to improve price transparency; and to advocate for appropriate national policies and strategies. Through series of improvements, this guideline has been used to measure medicine prices, availability, and affordability throughout the globe [16–18].

Ethiopia is one of the developing nations facing the dire consequences of unavailability and unaffordability of medicines [19]. To this end, studies conducted in the country are limited in the provision of region-specific price, availability and affordability data on EMs. Therefore, this study was designed to assess the price, availability and affordability of EMs in Eastern Ethiopia by using WHO/HAI survey methodology.

## Methods

### Study area, design and period

The study was conducted at public and private healthcare facilities in the major cities of Eastern Ethiopia including Dire Dawa, Harar, Haramaya, Chiro, Degahabour and Jigjiga. Dire Dawa is located 515 km east of Addis Ababa with a total area of 1288.02 km^2^. The area is dominated by dry, windy and hot climatic condition. According to the projections made based on the 2015 census, the total population of the administration is 383,529 of whom 283,773 (74%) live in urban part of the city. Harar is located 526 km away from Addis Ababa to the East. Harari Region is one of the nine National Regional States of Ethiopia, with the town of Harar as its capital. Based on the 2015 census, Harari had a total population of 183,344, of whom 92,258 were males and 99,321 or 54.17% of the population were urban inhabitants. Jigjiga is another major city in eastern Ethiopia, mainly inhabited by different Somali clans. Based on figures from the central statistics agency (CSA) in 2015, Jigjiga has an estimated total population of 250,000 of whom 126,578 were females. Haramaya is also one of the administrative centers in eastern Ethiopia located at a distance of 508 km from the capital, Addis Ababa. The 2015 national census reported a total population of 271,018, of whom 138,282 were males and 18.46% of its population were urban dwellers. Chiro, the capital of West Hararghe zone, is another major city in the eastern part of Ethiopia located at 373 km from Addis Ababa. The 2015 national census reported a total population for this town of 33,670, of whom 18,118 were males. Another city included in the study is Degehabur which is located in the Somali region of Ethiopia. Based on figures from the CSA in 2007, it had an estimated total population of 30,027 of whom 16,474 were males. A cross-sectional study was conducted from March 01- March 31, 2020.

### Population

All public and private healthcare facilities of Eastern Ethiopia were considered as a source population. Public and private healthcare facilities found in Eastern Ethiopia and which fulfilled criteria set forth by WHO/HAI guideline as well as essential medicines listed on WHO/HAI guideline and essential medicine list (EML) of Ethiopia were included for the study. Non-governmental health facilities, health facilities that provide medications free of cost and programmed essential medicines were excluded from this study.

### Sample size determination

Sixty medicine outlets were selected based on the WHO/HAI standardized sampling methodology [16]. Besides, WHO/HAI recommended that such price-based studies should include at least 14 global core medicines, and where possible 16 regional core medicines. Based on this, 30 drugs from global/regional core lists [16, 17] plus 20 other essential medicines from EML of Ethiopia [20] were included, making a total of 50 essential medicines per site, from different therapeutic classes for this study. This will enable the price and availability dynamics of the therapeutic group of medicines to be compared to those of other essential medicines which may be very meaningful in the interpretation of the findings and development of recommendation and strategies. It will also enable the publicly accessible global database of prices and availability to expand.

### Sampling procedure and technique

Based on the WHO/HAI standard sampling technique, six survey areas which cover a population of about 100,000 to 250,000, reachable within one day’s travel from the main urban center, large enough to represent the survey region and containing the requisite number of health facilities were selected [16, 17]. Based on this: Dire Dawa, Harar, Haramaya, Chiro, Degahabour and Jigjiga were selected as a survey area. In order to select the medicine outlets for the study based on WHO/HAI standard sampling technique, one main public hospital (referral, district or regional hospital) was selected from each survey area. Then, other four public health facilities, and five private medicine outlets, which are within 3-h travel from the main public hospital, were selected by using simple random sampling. In addition to the global and regional core list of essential medicines, 20 more essential medicines were randomly selected from EML of Ethiopia. The summary of generic name of essential medicines, strengths and unit of measurements, originator brands (OBs) and their respective manufacturers was presented in Table 1.

**Table 1:** List of essential medicines included in the analysis

Generic name	Dosage form and strength	Originator brand (OB)	Manufacturer
1. Salbutamol	Inhaler, 0.1 mg/dose	Ventoline	GSK
2. Metformin	Tablet, 500 mg	Glucophage	Bristol-Myers Squibb
3. Bisoprolol	Cap/tab, 5 mg	Concor	Merck
4. Captopril	Tab, 25 mg	Capoten	BMS
5. Simvastatin	Cap/tab, 20 mg	Zocor	MSD
6. Amitriptyline	Cap/tab, 25 mg	Tryptizol	MSD
7. Ciprofloxacin	Cap/tab, 500 mg	Ciproxin	Bayer
8. Co-trimoxazole	Suspension, 40 + 200 mg/5 ml	Bactrim	Roche
9. Amoxicillin	Cap/tab, 500 mg	Amoxil	GSK
10. Ceftriaxone	Injection, 1 g/vial	Rocephin	Roche
11. Diazepam	Cap/tab, 5 mg	Valium	Roche
12. Diclofenac	Cap/tab, 50 mg	Voltaren/cataflam	Novartis
13. Paracetamol	Suspension, 24 mg/ml	Panadol	GSK
14. Omeprazole	Cap/tab, 20 mg	Losec	Astra Zeneca
15. Glibenclamide	Cap/tab, 5 mg	Daonil	Sanofi-Aventis
16. Atenolol	Cap/tab, 50 mg	Tenormin	Astra Zeneca
17. Hyoscine Butylbromide	Tab, 10 mg	Buscopan	Sanofi-Aventis
18. Metoclopramide HCl	Tab, 10 mg	Maxolon	GSK
19. Bisacodyl	Tab, 5 mg	Dulcolax	Sanofi-Aventis
20. Oral rehydration salt	1L powder	–	–
21. Loperamide	Cap, 2 mg	Imodium	Johnson and Johnson
22. Amiodarone	Tab, 200 mg	Cordarone	Sanofi-Aventis
23. Furosemide	Tab, 40 mg	Lasix	Hoechst/Sanofi Aventis
24. Adrenaline	Injection, 0.1%, 1:1000 1 mg/ml	–	–
25. Paracetamol	Tab, 500 mg	Panadol	GSK
26. Acetylsalicylic Acid	Tab, 300 mg	Aspirin	Bayer
27. Acetylsalicylic Acid	Tab, 100 mg	Aspirin	Bayer
28. Ibuprofen	Tab, 400 mg	Brufen	Abbott
29. Fluoxetine	Cap, 20 mg	Prozac	Lilly
30. Sodium Valproate	Tab, 200 mg	Epilim	Sanofi-Aventis
31. Risperidone	Tab, 1 mg	Risperdal	Jansen Cilag
32. Haloperidol	Tab, 5 mg (0.5 mg)	Haldol	Jansen Cilag
33. Carbamazepine	Tab, 200 mg	Tegretol	Novartis
34. Allopurinol	Tab, 100 mg	Zyloprim	Mylan
35. Amoxicillin	Cap, 500 mg	Amoxil	GSK
36. Ampicillin	Injection (Sodium), 500 mg in vial	Totapen	BMS
37. Cloxacillin Sodium	Cap, 500 mg	Orbenine	Pfizer
38. Penicillin G Benzanthine	Injection 2.4, MIU In Vial	Penadur	Wyeth
39. Azithromycin	Cap, 250 mg	Zithromax	Pfizer
40. Ceftazidime	Injection 1 g in vial	Fortum	GSK
41. Doxycycline	Cap, 100 mg	Vibramycin	Pfizer
42. Metronidazole	Cap, 250 mg	Flagyl	Sanofi-Aventis
43. Co-trimoxazole	Tab, 400 mg + 80 mg	Bactrim	Roche
44. Fluconazole	Tab, 200 mg	Diflucan	Pfizer
45. Clindamycin	Cap, 150 mg	Cleocin	Pfizer
46. Hydrocortisone	Injection 50 mg/ml in 2 ml ampoule	Solu-Cortef	Pfizer
47. Propylthiouracil	Tab, 50 mg	–	–
48. Ferrous sulphate + Folic Acid	Cap, 200 mg + 0.5 mg	–	–
49. Tetracycline HCl	Eye ointment, 1%	Aureomycine	Frilab
50. Albendazole	Tab, 200 mg	Zentel	GSK

### Data collection methods

A standardized data collection tool developed by WHO/HAI, with necessary modifications was employed to collect the data from both public and private facilities. The data were collected by twelve pharmacists who were recruited as data collectors. The data regarding price and availability of essential medicines were collected from each selected drug retail outlet.

### Study variables

The availability, price and affordability of essential medicines were considered as the outcome variables. Type of sector, source of drugs (local or imported), the nature of facilities, duration of therapy, monthly income of lowest paid government worker (to be converted to daily wage), the type of medicines (OBs and LPGs) were treated as independent variables.

### Data processing and analysis

After data collection, data were entered and analyzed using Excel® WHO/HAI Medicine Pricing Workbook and the results were summarized and presented in tables and graphs. Medicine availability was calculated as percent availability of individual medicines; mean (average) percent (%) availability across a group of medicines; and variations between product types (OBs vs LPGs) and sectors. For further statistical analysis, the data were transferred to SPSS version 20. Normality distribution of the price data was checked using Kolmogorov–Smirnov and Shapiro–Wilk tests. Accordingly, the Wilcoxon–Mann–Whitney *U* test was employed to compare the median buyers’ price (customers’ out-of-pocket expenditure for drugs) between public and private health facilities. Kruskal–Wallis test was also run to explore the median price difference among four facilities (hospital, health center, pharmacy and drug store). Medicine prices were calculated as median prices of individual medicines in United States Dollar (USD). The exchange rate of Ethiopian birr to USD equivalent was considered by taking the monthly average of March, 2020 (1 USD = 35.70 Ethiopian birr).; median price ratio (MPR) was computed as ratios of median local price to international (WHO/HAI) buyers’ reference price for public, private and overall facilities as follows.

$${\text{MPR}} = \frac{{{\text{Median}}\;{\text{price}}\;{\text{of}}\;{\text{a}}\;{\text{given}}\;{\text{drug}}\;{\text{(USD)}}}}{{{\text{International}}\;{\text{buyers'}}\;{\text{median}}\;{\text{price}}\;{\text{of}}\;{\text{that}}\;{\text{drug}}\;{\text{(USD)}}}}$$

Treatment affordability was calculated based on the daily wage of the lowest-paid government employee; and components of the prices of medicines paid by consumers. Daily wage of the lowest paid government worker of Ethiopia was about 0.44 USD (https://mywage.org/ethiopia/labour-law/wages). Accordingly, the affordability was also computed for public and private sectors for ease of comparison. Affordability (in terms of the number of daily wages) was computed as follows:

$${\text{Affordability}} = \frac{{{\text{The~total~price~of~the~regimen~for~a~given~drug~~}}\left( {{\text{USD}}} \right)}}{{{\text{The~daily~wage~of~lowest~paid~government~employee~}}\left( {{\text{USD}}} \right)}}$$

## Result

### Availability of essential medicines

In 60 health facilities surveyed, nearly half (*n* = 26) of the OB versions of essential medicines were not available at all during the study. From which, 6 OB medicines out of 14 WHO/HAI core drugs were not available at all. Besides, only four OB essential medicines (glibenclamide 5 mg, paracetamol 500 mg, carbamazepine 200 mg and acetyl salicylic acid (ASA) 100 mg tablets) were available in more than 10% of the settings surveyed. The overall (pooled) percent availability of OB versions of these essential medicines in all facilities (both public and private) was about 3.6% (range: 0–31.7%). Observing the public medicine outlets alone, 39 OB medicines (78%) were not available in all facilities during the study period. Moreover, except carbamazepine/Tegretol (*n* = 7) and azithromycin/Zithromax (*n* = 4), the rest drugs were available in only one of the 28 public medicine outlets surveyed. The overall percent availability of OB medicines in surveyed public sectors was 1.43%. Regarding the private sector, 30 OB medicines (60%) were not available at all. Only 6 OB versions of drugs (metformin 500 mg, diclofenac 50 mg, glibenclamide 5 mg, paracetamol 500 mg, ASA 100, and carbamazepine 200 mg tabs) were available in more than 10% of the private facilities surveyed. What is more, the OB versions of drugs like paracetamol suspension, diclofenac 50 tab, glibenclamide 5 mg tab, salbutamol inhaler, ibuprofen 400 tab and ASA 100 tab were available in private sectors only. The overall availability of OB versions of 50 essential medicines in private sector was about 5.50% with pharmacy and drug store contributing 6.1% and 4.5%, respectively (Table 2).

**Table 2:** Availability of essential medicine (OB and LPGs) based on WHO/HAI methodology

Drugs (names, strengths and units)	Availability of essential medicines													
	Originator brand (*n* = 60)							Lowest price generics (*n* = 60)						
	Public facilities (%)		Total (*n* = (28)	Private facilities (%)		Total (*n* = 32)	Overall (*N* = 60)	Public facilities (%)		Total (*n* = 28)	Private facilities (%)		Total (*n* = 32)	Overall (*N* = 60)
	Hospital (*n* = 9)	HC (*n* = 19)		Pharmacy (*n* = 20)	DS (*n* = 12)			Hospital (*n* = 9)	HC (*n* = 19)		Pharmacy (*n* = 20)	DS (*n* = 12)		
Salbutamol 0.1 mg/dose	0	0	0	0	3	3	3 (5.0)	8	7	15	19	4	23	38 (63.3)
Metformin 500 mg tab	0	1	1	4	0	4	5 (8.3)	8	14	22	18	10	28	50 (83.3)
Bisoprolol 5 mg/tab	0	0	0	1	0	1	1 (1.7)	0	0	0	0	1	1	1 (1.7)
Captopril 25 mg tab	0	1	1	0	0	0	1 (1.7)	5	2	7	3	0	3	10 (16.7)
Simvastatin 20 mg tab/cap	0	0	0	0	0	0	0	0	0	0	6	1	7	7 (11.7)
Amitriptyline 25 mg tab/cap	0	0	0	0	0	0	0	3	4	7	13	9	22	29 (48.3)
Cipro 500 mg tab/cap	1	0	1	0	0	0	1 (1.7)	8	12	20	19	11	30	50 (83.3)
Cotri-mox 240/5 susp	0	0	0	0	0	0	0	3	9	12	19	12	31	43 (71.7)
Amox 500 mg cap/tab	0	0	0	0	0	0	0	8	18	26	19	11	30	56 (93.3)
Ceftriaxone 1 g vial	0	0	0	0	0	0	0	5	17	22	19	12	31	53 (88.3)
Diazepam 5 mg tab/cap	0	1	1	0	0	0	1 (1.7)	7	8	15	4	1	5	20 (33.3)
Diclofenac 50 mg tab/cap	0	0	0	4	1	5	5 (8.3)	6	14	20	18	10	28	48 (80.0)
Paracetamol 24 mg/ml susp	0	0	0	2	0	2	2 (3.3)	2	5	7	15	10	25	32 (53.3)
Omeprazole 20 mg tab/cap	0	0	0	1	0	1	1 (1.7)	7	15	22	20	12	32	54 (90.0)
Glibenclamide 5 mg tab/cap	0	0	0	13	6	19	19 (31.7)	9	13	22	14	3	17	39 (65.0
Atenolol 50 mg tab/cap	0	0	0	0	0	0	0	5	3	8	18	5	23	31 (51.7)
Hyoscine BB 10 mg tab	0	0	0	0	0	0	0	8	14	22	16	6	22	44 (73.3
Metoclopramide HCl 10 mg tab	1	0	1	0	0	0	1 (1.7)	8	12	20	16	8	24	44 (73.3)
Bisacodyl 5 mg tab	0	0	0	0	0	0	0	6	9	15	13	9	22	37 (61.7)
ORS sack (1 L)	0	0	0	2	0	2	2 (3.3)	6	12	18	19	10	29	47 (78.3)
Loperamide 2 mg cap	0	0	0	0	0	0	0	0	0	0	2	1	3	3 (5.0)
Amiodarone 200 mg tab	0	0	0	0	0	0	0	0	0	0	0	1	1	1 (1.7)
Furosemide 40 mg tab	0	1	1	2	1	3	4 (6.7)	8	10	18	19	9	28	46 (76.7)
Adrenaline 1 mg/ml inj	0	0	0	0	0	0	0	7	11	18	4	0	4	22 (36.7)
Paracetamol 500 mg tab	0	1	1	10	6	16	17 (28.3)	7	13	20	19	7	26	46 (76.7)
ASA 300 mg tab	0	0	0	0	0	0	0	6	6	12	9	3	12	24 (40.0)
ASA 100 mg tab	0	0	0	7	1	8	8 (13.3)	0	0	0	2	3	5	5 (8.3)
Ibuprofen 400 mg tab	0	0	0	1	2	3	3 (5.0)	5	12	17	18	9	27	44 (73.3)
Fluoxetine 20 mg cap	0	0	0	0	0	0	0	6	4	10	7	1	8	18 (30.0)
Valproate 200 mg tab	0	0	0	3	0	3	3 (5.0)	4	1	5	5	0	5	10 (16.7)
Risperidone 1 mg tab	0	0	0	0	0	0	0	5	1	6	1	0	1	7 (11.7)
Haloperidol 5 (0.5) mg tab	0	0	0	0	0	0	0	5	1	6	4	0	4	10 (16.67)
Carbamazepine 200 mg tab	5	2	7	9	3	12	19 (31.7)	0	0	0	2	0	2	2 (3.3)
Allopurinol 100 mg tab	0	0	0	0	0	0	0	2	2	4	4	0	4	8 (13.3)
Amox 250 mg cap	0	0	0	0	0	0	0	8	15	23	12	7	19	42 (70)
Ampicillin 500 mg inj	0	0	0	0	1	1	1 (1.7)	6	5	11	11	5	16	27 (45.0)
Cloxa 500 mg cap/tab	0	0	0	0	0	0	0	5	10	15	11	7	18	33 (55.0)
Beza Pen G 2.4 MIU	0	0	0	0	0	0	0	3	5	8	4	0	4	12 (20.0)
Azithromycin 250 mg cap	1	3	4	1	1	2	6 (10.0)	1	2	3	8	4	12	15 (25.0)
Ceftazidime 1 g inj	0	0	0	0	0	0	0	2	0	2	6	2	8	10 (16.7)
Doxycycline 100 mg cap	0	0	0	0	0	0	0	9	16	25	19	9	28	53 (88.3)
Metronidazole 250 mg caps	0	0	0	0	1	1	1 (1.7)	7	15	22	20	8	28	50 (83.3)
Cotri-mox 480 mg tabs	0	0	0	0	0	0	0	2	12	14	16	5	21	35 (58.3)
Fluconazole 200 mg tab	0	0	0	0	0	0	0	5	3	8	9	3	12	20 (33.3)
Clindamycin 150 mg	0	0	0	0	0	0	0	2	0	2	2	0	2	4 (6.7)
Hydrocortisone (50 mg/ml) inj	0	0	0	0	0	0	0	6	4	10	14	7	21	31 (51.7)
PTU 50 mg tab	0	0	0	0	0	0	0	5	0	5	3	1	4	9 (15.0)
Fefol 200 mg + 0.5 mg cap	0	0	0	1	0	1	1 (1.7)	5	4	9	15	6	21	30 (50.0)
TTC 1% ointment	0	1	1	0	1	1	2 (3.3)	4	10	14	13	7	20	34 (56.7)
Albendazole 200 mg tab	0	1	1	0	0	0	1 (1.7)	2	6	8	10	7	17	25 (41.7)
Total	8	12	20	61	27	88	108	239	356	595	557	257	814	1409
Maximum expected	450	950	1400	1000	600	1600	3000	450	950	1400	1000	600	1600	3000
Overall average % (pooled)	1.78	1.26	1.43	6.10	4.50	5.50	3.60	53.11	37.47	42.50	55.70	42.83	50.88	46.97

Regarding the LPG versions of these essential medicines, all the LPG versions of selected essential medicines were available at least in one of the surveyed health facilities. The overall percent availability of LPGs in all settings was 46.97%, ranging from 1.7% (bisoprolol 5 mg and amiodarone 200 mg tabs) to 93.3% (amoxicillin 500 mg cap). The LPG versions of six drugs (bisoprolol, simvastatin, loperamide, amiodarone, ASA 100 mg, and carbamazepine) were not available at all in public facilities and one of which was from the WHO/HAI core drug category. In general, 26 LPG versions (52%) of the surveyed medicines were available in 50% or more of the facilities included in the study. Only eight LPG versions were available in 80% or more of the facilities surveyed. In descending order, amoxicillin 500 mg caps (93.3%), omeprazole 20 mg cap (90%), ceftriaxone 1 g inj. vial (88.3%), doxycycline 100 mg cap (88.3%), metformin 500 mg tab (83.3%), ciprofloxacin 500 mg tab (83.3%), metronidazole 250 mg cap (83.3%), and diclofenac 50 mg tab (80.0%) were the top eight drugs available during the study. The overall percent availability of LPGs in surveyed public sectors was 42.5% (hospital = 53.11% and health center = 37.47%) whereas that of the private counterparts were 50.8% (pharmacy = 55.7% and drug store = 42.83%) (Table 2).

**Fig. 1:**
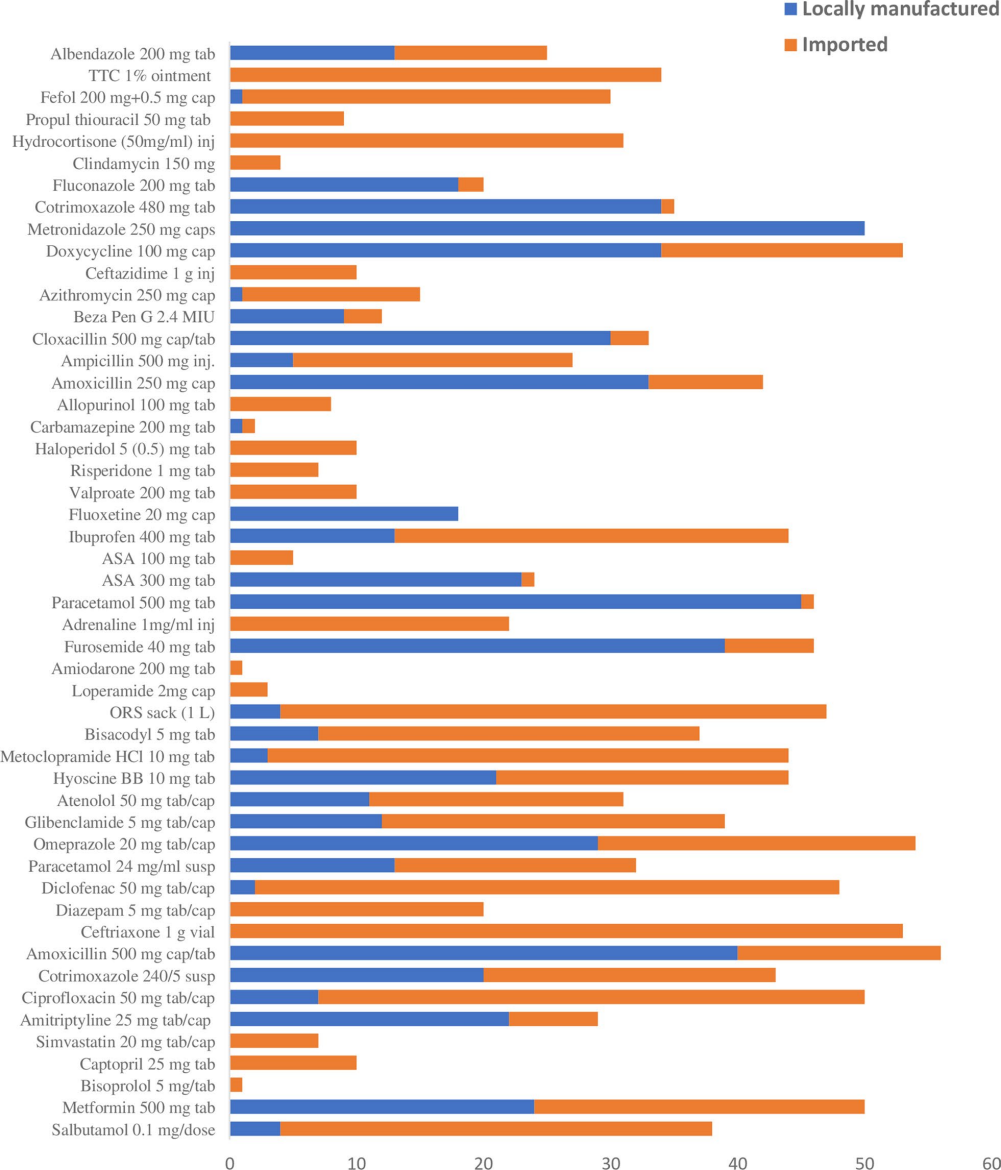
Availability based on the source of drugs

Regarding the source of available drugs, 17 LPGs (34%) were totally imported. From which, four drugs (diazepam, ceftriaxone, simvastatin and captopril) were among the WHO/HAI core list. Except ceftriaxone (*n* = 53), TTC (*n* = 34) and hydrocortisone (*n* = 31), all other imported drugs (LPG versions) were available in less than 50% of the surveyed facilities. Two drugs (metronidazole and fluoxetine) were from local source only. The rest 31 drugs were from both sources with certain domination from imported ones (Fig. 1).

**Fig. 2:**
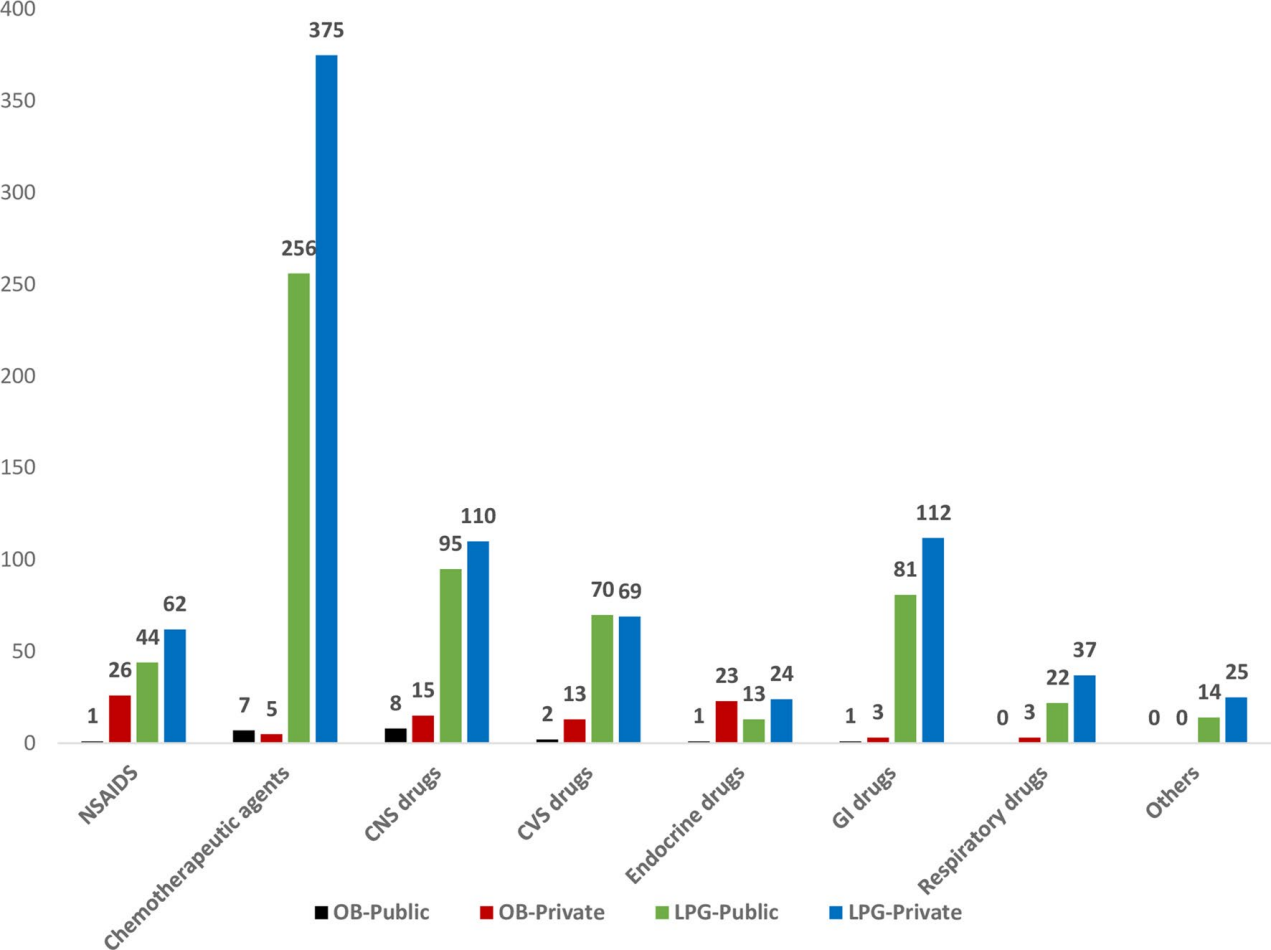
Availability by therapeutic class of LPG versions of essential medicines

Regarding the therapeutic classes, the study included chemotherapeutic agents (*n* = 17), cardiovascular drugs (CVS) (*n* = 8), central nervous system (CNS) drugs (*n* = 7), gastrointestinal drugs (*n* = 6), non-steroidal anti-inflammatory drugs (NSAIDs) (*n* = 5), respiratory agents (*n* = 2) and two more from miscellaneous agents. Comparing the overall availability, the LPG versions of these drugs were obtained from private settings in almost all therapeutic classes. OB medicines obtained from public facilities were primarily from CNS (40%) and chemotherapeutic drugs (35%) whereas those obtained from private counterparts were from NSAIDs (29.5%) and endocrine agents (26.1%). Generally, the average percent availability per class indicated that the top three available (for any LPG versions) were chemotherapeutic drugs (public: 53.78%; private: 68.93%), CNS drugs (public: 48.47%; private: 49.11%) and GI agents (public: 48.21%; private: 58.33%) (Fig. 2).

**Fig. 3:**
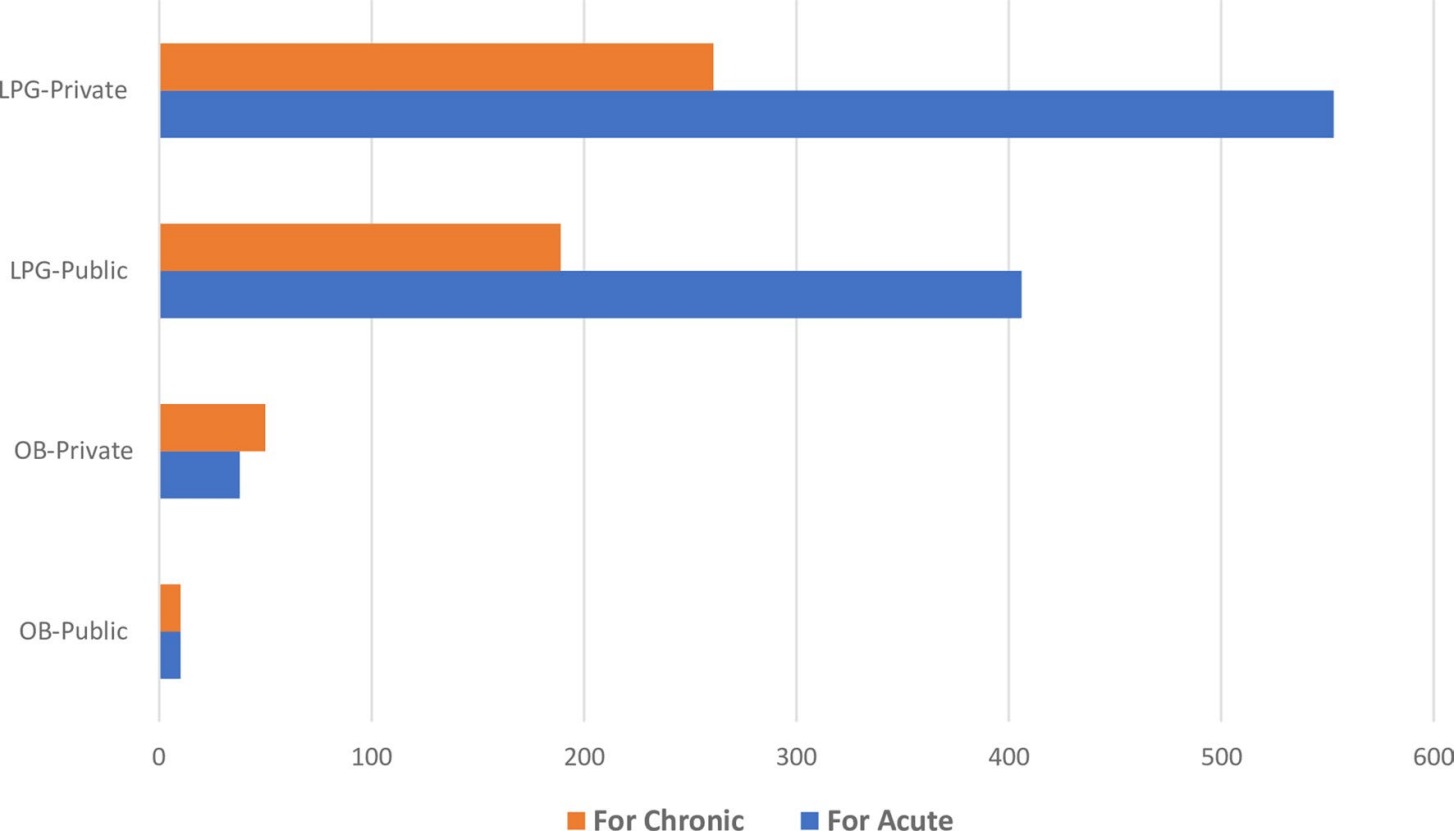
Availability based on the duration of therapy of LPG versions of essential medicines

Based on the duration of therapeutic regimen, majority of the drugs available in both public and private settings were those agents being used for acute conditions (for less than 2 weeks). The average percent availability of drugs used for acute and chronic conditions was 51.26% (public: 46.77%; private: 55.75%) and 39.23% (public: 35.53%; private: 42.93%), respectively (Fig. 3).

### Cost analysis of essential medicines

Drugs like adrenaline, ASA 300, and ibuprofen had comparable median buyers’ price between public and private medicine outlets. On the other hand, the median buyers’ price of drugs in private settings were higher than the public counterparts for 94% (*n* = 47) of LPGs analyzed. From these, the Mann–Whitney *U* test indicated that 64% (*n* = 32) drugs showed statistically significant median price difference between public and private settings (*p* < 0.05) (Table 3). Specifically, the private median prices of LPG versions were more than three times that of the public sector for drugs such as ampicillin, azithromycin, ceftazidime, diazepam, fluconazole, hydrocortisone, metoclopramide and ORS. Likewise, Kruskal–Wallis test indicated that 50% of drugs showed statistically significant median price difference across facilities (Table 4).

**Table 3:** Median price of LPG versions of essential medicines (USD) by ownership

Name of the drugs (name, strength and Unit)	Lowest price generics (LPG)								*p *value
	Public facilities Median price, USD			Private facilities (%) Median price, USD			Mann–Whitney *U*	Wilcoxon W	
	Median	25th	75th	Median	25th	75th			
1. Salbutamol 0.1 mg/dose	2.062	1.888	2.092	3.385	3.077	4.000	19.5	139.5	0.000*
2. Metformin 500 mg tab	0.022	0.017	0.031	0.031	0.031	0.046	87	340	0.000*
3. Bisoprolol 5 mg/tab	–	–	–	0.077	0.077	0.077	–	–	–
4. Captopril 25 mg tab	0.031	0.019	0.031	0.046	0.025	–	6	34	0.298
5. Simvastatin 20 mg tab/cap	–	–	–	0.206	0.086	0.236	–	–	–
6. Amitriptyline 25 mg tab/cap	0.019	0.019	0.031	0.054	0.041	0.077	17	45	0.002*
7. Cipro 50 mg tab/cap	0.031	0.031	0.046	0.062	0.046	0.077	73.5	283.5	0.000*
8. Cotri-mox 240/5 susp	0.873	0.638	0.954	0.923	0.923	1.077	126.5	204.5	0.102
9. Amox 500 mg cap/tab	0.031	0.031	0.031	0.046	0.040	0.062	75	426	0.000*
10. Ceftriaxone 1 g vial	0.615	0.546	0.769	0.769	0.615	0.800	221	474	0.028*
11. Diazepam 5 mg tab/cap	0.012	0.006	0.031	0.040	0.007	0.077	20.5	140.5	0.134
12. Diclofenac 50 mg tab/cap	0.007	0.006	0.015	0.015	0.009	0.031	127.5	337.5	0.001*
13. Paracetamol 24 mg/ml susp	0.462	0.308	0.985	0.923	0.769	0.923	61.5	89.5	0.222
14. Omeprazole 20 mg tab/cap	0.016	0.015	0.019	0.031	0.022	0.031	105	358	0.000*
15. Glibenclamide 5 mg tab/cap	0.009	0.008	0.031	0.022	0.017	0.042	87	340	0.004*
16. Atenolol 50 mg tab/cap	0.023	0.015	0.031	0.031	0.028	0.046	46.5	82.5	0.035*
17. Hyoscine BB 10 mg tab	0.105	0.092	0.108	0.111	0.099	0.123	167.5	443.5	0.032*
18. Metoclopramide HCl 10 mg tab	0.009	0.006	0.015	0.031	0.015	0.031	85	295	0.000*
19. Bisacodyl 5 mg tab	0.023	0.021	0.031	0.062	0.031	0.077	27.5	147.5	0.000*
20. ORS sack (1 L)	0.080	0.000	0.154	0.308	0.215	0.308	51.5	241.5	0.000*
21. Loperamide 2 mg cap	–	–	–	0.292	0.092	–	–	–	–
22. Amiodarone 200 mg tab	–	–	–	–	–	–	–	–	–
23. Furosemide 40 mg tab	0.020	0.012	0.030	0.031	0.031	0.041	81.5	252.5	0.000*
24. Adrenaline 1 mg/ml inj	0.138	0.113	0.162	0.138	0.092	0.623	34	44	0.863
25. Paracetamol 500 mg tab	0.006	0.006	0.015	0.015	0.015	0.019	90	300	0.000*
26. ASA 300 mg tab	0.015	0.014	0.031	0.015	0.010	0.015	53	131	0.258
27. ASA 100 mg tab	–	–	–	0.092	0.042	0.231	–	–	–
28. Ibuprofen 400 mg tab	0.031	0.025	0.031	0.031	0.031	0.031	137.5	290.5	0.003*
29. Fluoxetine 20 mg cap	0.032	0.031	0.035	0.095	0.081	0.119	9	64	0.006*
30. Valproate 200 mg tab	0.078	0.051	0.092	0.123	0.104	0.154	1	16	0.015*
31. Risperidone 1 mg tab	0.208	0.044	0.222	0.280	0.280	0.280	0	21	0.134
32. Haloperidol 5 (0.5) mg tab	0.023	0.018	0.031	0.062	0.034	–	0	21	0.019*
33. Carbamazepine 200 mg tab	–	–	–	0.088	0.062	–	–	–	–
34. Allopurinol 100 mg tab	0.032	0.031	0.034	0.060	0.036	0.158	1	11	0.037*
35. Amox 250 mg cap	0.015	0.015	0.029	0.031	0.031	0.062	67.5	367.5	0.000*
36. Ampicillin 500 mg inj	0.154	0.123	0.246	0.462	0.308	0.615	30	96	0.004*
37. Cloxa 500 mg cap/tab	0.045	0.031	0.051	0.062	0.046	0.082	45	136	0.006*
38. Beza Pen G 2.4 MIU	0.280	0.223	0.308	0.462	0.308	1.077	5	41	0.053
39. Azithromycin 250 mg cap	0.123	0.123	0.123	0.462	0.279	0.590	6	12	0.081
40. Ceftazidime 1 g inj	0.846	0.831		2.923	2.592	3.077	0.5	3.5	0.047*
41. Doxycycline 100 mg cap	0.019	0.017	0.026	0.031	0.031	0.046	75.5	400.5	0.000*
42. Metronidazole 250 mg caps	0.015	0.012	0.028	0.017	0.015	0.031	209.5	462.5	0.048*
43. Cotri-mox 480 mg tabs	0.015	0.012	0.016	0.031	0.015	0.031	66.5	171.5	0.005*
44. Fluconazole 200 mg tab	0.037	0.032	0.039	0.169	0.108	0.215	6	42	0.001*
45. Clindamycin 150 mg	0.129	0.128	–	0.097	0.092	–	0	3	0.121
46. Hydrocortisone (50 mg/ml) inj	0.492	0.492	0.869	1.846	1.308	2.154	10.5	65.5	0.000*
47. PTU 50 mg tab	0.145	0.106	0.149	0.269	0.132	0.294	4	19	0.140
48. Fefol 200 mg + 0.5 mg cap	0.028	0.016	0.031	0.055	0.046	0.062	8	53	0.000*
49. TTC 1% ointment	0.185	0.154	0.308	0.308	0.308	0.308	50.5	155.5	0.001*
50. Albendazole 200 mg tab	0.031	0.031	0.154	0.077	0.031	0.154	46	74	0.388

Wilcoxon–Mann–Whitney *U* test for two independent groups (public and private settings) with skewed price distribution

**Table 4:** Median price of LPGs (USD) by facilities (Kruskal–Wallis Test)

	Median price in USD												Chi-Squared (KWT)	*p*-value
Drugs, strength and unit	Hospital			Health center			Pharmacy			Drug store				
	Median	25th	75th	Median	25th	75th	Median	25th	75th	Median	25th	75th		
Salbutamol 0.1 mg/dose	2.0769	1.9946	2.6923	1.8877	1.7846	2.0646	3.3846	3.0769	4.0000	3.3846	3.0769	3.9231	21.97	.000*
Metformin 500 mg tab	0.0185	0.0158	0.0262	0.0239	0.0177	0.0308	0.0308	0.0273	0.0462	0.0462	0.0308	0.0646	21.62	.000*
Bisoprolol 5 mg/tab	–	–	–	–	–	–	–	–	–	0.0769	0.0769	0.0769	–	–
Captopril 25 mg tab	0.0215	0.0185	0.1093	0.0308	0.0308	0.0308	0.0462	0.0246	–	–	–	–	1.50	.473
Simvastatin 20 mg tab/cap	–	–	–	–	–	–	0.2031	0.0851	0.2383	0.2308	0.2308	0.2308	0.25	.617
Amitriptyline 25 mg tab/cap	0.0308	0.0185	–	0.0185	0.0173	0.0277	0.0462	0.0365	0.0615	0.0769	0.0539	0.0923	15.65	.001*
Cipro 50 mg tab/cap	0.0308	0.0308	0.0412	0.0319	0.0308	0.0577	0.0615	0.0462	0.0769	0.0646	0.0615	0.0923	21.51	.000*
Cotri-mox 240/5 susp	0.7692	0.6154	–	0.8846	0.6462	1.0154	0.9231	0.8615	1.0769	0.9231	0.9231	1.1923	4.66	.198
Amox 500 mg cap/tab	0.0308	0.0308	0.0308	0.0308	0.0308	0.0338	0.0462	0.0400	0.0523	0.0462	0.0400	0.0615	30.01	.000*
Ceftriaxone 1 g vial	0.5538	0.3847	0.6923	0.6154	0.5538	0.7692	0.7692	0.5538	0.8000	0.7692	0.6154	0.8846	7.30	.063
Diazepam 5 mg tab/cap	0.0154	0.0062	0.0308	0.0093	0.0059	0.0270	0.0508	0.0147	0.0846	0.0077	0.0077	0.0077	4.45	.216
Diclofenac 50 mg tab/cap	0.0093	0.0058	0.0154	0.0067	0.0062	0.0154	0.0154	0.0092	0.0923	0.0154	0.0139	0.0169	10.84	.013*
Paracetamol 24 mg/ml susp	0.3846	0.3077	–	0.9231	0.3000	1.2616	0.9231	0.7692	0.9231	0.9231	0.6154	0.9231	4.59	.205
Omeprazole 20 mg tab/cap	0.0154	0.0154	0.0185	0.0172	0.0123	0.0308	0.0308	0.0215	0.0308	0.0308	0.0246	0.0308	20.86	.000*
Glibenclamide 5 mg tab/cap	0.0092	0.0070	0.0123	0.0154	0.0083	0.0308	0.0215	0.0185	0.0462	0.0154	0.0154		11.13	.011*
Atenolol 50 mg tab/cap	0.0185	0.0154	0.0370	0.0308	0.0154		0.0308	0.0269	0.0392	0.1538	0.0308	0.1538	8.44	.038*
Hyoscine BB 10 mg tab	0.0985	0.0923	0.1193	0.1046	0.0738	0.1077	0.1108	0.1031	0.1231	0.1154	0.0846	0.1231	4.80	.187
Metoclopramide HCl 10 mg tab	0.0062	0.0039	0.0139	0.0092	0.0062	0.0154	0.0308	0.0154	0.0331	0.0154	0.0154	0.0308	15.10	.002*
Bisacodyl 5 mg tab	0.0223	0.0200	0.0254	0.0277	0.0199	0.0308	0.0615	0.0385	0.0769	0.0615	0.0308	0.0923	18.72	.000*
ORS sack (1 L)	0.1077	0.0000	0.1861	0.0797	0.0000	0.1538	0.3077	0.2154	0.3077	0.3077	0.2000	0.3077	23.74	.000*
Loperamide 2 mg cap	–	–	–	–	–	–	0.1923	0.0923		0.4615	0.4615	0.4615	1.50	.221
Amiodarone 200 mg tab	–	–	–	–	–	–	–	–	–	–	–	–	–	–
Furosemide 40 mg tab	0.0185	0.0123	0.0293	0.0231	0.0122	0.0296	0.0308	0.0308	0.0415	0.0308	0.0308	0.0416	15.79	.001*
Adrenaline 1 mg/ml inj	0.1538	0.1231	0.7692	0.1231	0.1108	0.1538	0.1385	0.0923	0.6231	–	–	–	1.44	.486
Paracetamol 500 mg tab	0.0123	0.0062	0.0154	0.0062	0.0062	0.0154	0.0154	0.0154	0.0185	0.0154	0.0154	0.0215	15.51	.001*
ASA 300 mg tab	0.0154	0.0119	0.0177	0.0308	0.0131	0.0476	0.0154	0.0108	0.0154	0.0154	0.0092	–	3.79	.285
ASA 100 mg tab	–	–	–	–	–	–	0.0422	0.0166	–	0.0923	0.0923	–	3.16	.076
Ibuprofen 400 mg tab	0.0308	0.0285	0.0308	0.0308	0.0223	0.0308	0.0308	0.0308	0.0308	0.0308	0.0308	0.0308	10.16	.017*
Fluoxetine 20 mg cap	0.0323	0.0308	0.0346	0.0331	0.0146	0.0354	0.0923	0.0769	0.1231	0.1077	0.1077	0.1077	7.99	.046*
Valproate 200 mg tab	0.0769	0.0454	0.0923	0.0775	0.0775	0.0775	0.1231	0.1039	0.1538	–	–	–	5.96	.051
Risperidone 1 mg tab	0.2154	0.1193	0.2231	0.0462	0.0462	0.0462	0.2800	0.2800	0.2800	–	–	–	2.83	.243
Haloperidol 5 (0.5) mg tab	0.0231	0.0170	0.0308	0.0225	0.0225	0.0225	0.0615	0.0338	–	–	–	–	5.53	.063
Carbamazepine 200 mg tab	–	–	–	–	–	–	0.0885	0.0615	–	–	–	–	–	–
Allopurinol 100 mg tab	0.0338	0.0338	0.0338	0.0308	0.0308	0.0308	0.0600	0.0361	0.1577	–	–	–	5.45	.066
Amox 250 mg cap	0.0154	0.0154	0.0154	0.0195	0.0154	0.0308	0.0308	0.0246	0.0308	0.0615	0.0369	0.0615	24.70	.000*
Ampicillin 500 mg inj	0.1538	0.1193	0.1769	0.1538	0.1160	0.3677	0.3077	0.1538	0.6154	0.4615	0.3846	0.5385	9.26	.026*
Cloxa 500 mg cap/tab	0.0308	0.0231	0.0447	0.0462	0.0388	0.0600	0.0646	0.0457	0.0808	0.0615	0.0462	0.0923	10.49	.015*
Beza Pen G 2.4 MIU	0.3077	0.2154	–	0.2523	0.2216	0.3077	0.4616	0.3077	1.0770	–	–	–	4.33	.115
Azithromycin 250 mg cap	0.1231	0.1231	0.1231	0.1231	0.1231	0.1231	0.4615	0.3512	0.9223	0.2539	0.0347	0.5001	5.35	.148
Ceftazidime 1 g inj	0.8462	0.8308	–	–	–	–	2.9231	2.1538	3.3077	2.8462	2.6154	–	3.96	.138
Doxycycline 100 mg cap	0.0185	0.0159	0.0216	0.0185	0.0169	0.0300	0.0308	0.0234	0.0369	0.0308	0.0308	0.0462	25.02	.000*
Metronidazole 250 mg caps	0.0123	0.0123	0.0246	0.0154	0.0123	0.0308	0.0185	0.0154	0.0308	0.0154	0.0154	0.0270	5.90	.117
Cotri-mox 480 mg tabs	0.0139	0.0092	–	0.0154	0.0123	0.0154	0.0247	0.0154	0.0308	0.0308	0.0154	0.0385	8.21	.042*
Fluconazole 200 mg tab	0.0369	0.0354	0.0508	0.0308	0.0308		0.1692	0.0769	0.2154	0.1538	0.1538	–	11.42	.010*
Clindamycin 150 mg	0.1293	0.1277	–	–	–	–	0.0974	0.0923	–	–	–	–	2.40	.121
Hydrocortisone (50 mg/ml) inj	0.4923	0.4923	0.8692	0.6308	0.4923	1.1154	1.6154	1.2308	2.1538	1.8462	1.3846	3.0769	17.06	.001*
PTU 50 mg tab	0.1446	0.1062	0.1492	–	–	–	0.2769	0.0892		0.2615	0.2615	0.2615	2.22	.329
Fefol 200 mg + 0.5 mg cap	0.0277	0.0154	0.0308	0.0247	0.0173	0.0361	0.0554	0.0462	0.0615	0.0585	0.0431	0.1253	15.83	.001*
TTC 1% ointment	0.1846	0.1615	0.2769	0.1846	0.1500	0.3077	0.3077	0.3077	0.3692	0.3077	0.3077	0.3077	12.07	.007*
Albendazole 200 mg tab	0.0231	0.0154	–	0.0378	0.0308	0.1692	0.0739	0.0250	0.1538	0.0769	0.0615	0.3077	3.38	.336

Regarding the WHO/MSH median buyers’ price, the MPR indicated that the median prices of drugs in public facilities were more than three times the reference price in 8 LPG versions of essential medicines including atenolol, captopril, fluoxetine, furosemide, cotrimoxazole suspension, paracetamol suspension, salbutamol inhaler, and risperidone tablets. Drugs like metronidazole, propylthiouracil, ibuprofen, and hyoscine had local buyers’ price of more than two times the international median price. Looking at the private sectors, the MPR value indicated that the median buyers’ price of drugs were more than four times the international reference price in 30% of drugs. Overall, drugs with top ten MPR were salbutamol inhaler, cotrimoxazole suspension, paracetamol suspension, loperamide tab, ASA 100, simvastatin, fluoxetine, risperidone, atenolol and furosemide (Table 5).

**Table 5:** Overall median price, median price ratios (MPR) and affordability of LPGs based on WHO/MSH reference guide (buyers’ price)

Name of the drugs	Lowest price generics (LPG)							Private to public ratio
	Overall Median price (USD)	25th	75th	WHO/MSH buyers’ median price (USD)	MPR for public facilities	MPR for private facilities	Overall MPR	
Adrenaline 1 mg/ml inj	0.1385	0.1062	0.1846	0.1926	0.72	0.72	0.72	1.00
Albendazole 200 mg tab	0.0615	0.0308	0.1538	0.0328	0.95	2.35	1.88	2.48
Allopurinol 100 mg tab	0.0338	0.0316	0.0685	–	–	–	–	1.88
Amiodarone 200 mg tab	–	–	–	–	–	–	–	–
Amitriptyline 25 mg tab/cap	0.0462	0.0308	0.0739	0.0281	0.68	1.92	1.64	2.84
Amox 250 mg cap	0.0246	0.0154	0.0308	0.0227	0.66	1.37	1.08	2.07
Amox 500 mg cap/tab	0.0369	0.0308	0.0462	0.0299	1.04	1.54	1.23	1.48
Ampicillin 500 mg inj	0.3077	0.1538	0.4615	0.3696	0.42	1.25	0.83	3.00
ASA 100 mg tab	0.0923	0.0422	0.2308	0.0062	–	14.84	14.89	–
ASA 300 mg tab	0.0154	0.0123	0.0293	0.0391	0.38	0.38	0.39	1.00
Atenolol 50 mg tab/cap	0.0308	0.0246	0.0462	0.0059	3.90	5.25	5.22	1.35
Azithromycin 250 mg cap	0.3591	0.1231	0.5129	0.198	0.62	2.33	1.81	3.76
Beza Pen G 2.4 MIU	0.3077	0.2477	0.3308	–	–	–	–	1.65
Bisacodyl 5 mg tab	0.0308	0.0239	0.0615	0.0147	1.56	4.22	2.10	2.70
Bisoprolol 5 mg/tab	–	–	–	0.0462	–	1.67	1.67	–
Captopril 25 mg tab	0.0308	0.0208	0.0654	0.0076	4.08	6.05	4.05	1.48
Carbamazepine 200 mg tab	0.0885	0.0461	0.0922	0.0202	–	4.36	4.38	–
Ceftazidime 1 g inj	2.6923	0.8615	3.0769	1.77	0.48	1.65	1.52	3.46
Ceftriaxone 1 g vial	0.6462	0.5538	0.7692	0.4251	1.45	1.81	1.52	1.25
Cipro 500 mg tab/cap	0.0615	0.0308	0.0685	0.0269	1.15	2.30	2.29	2.00
Clindamycin 150 mg	0.1151	0.0949	0.1300	0.173	0.75	0.56	0.67	0.75
Cloxa 500 mg cap/tab	0.0462	0.0438	0.0708	0.0566	0.80	1.10	0.82	1.38
Cotri-mox 240/5 susp	0.9231	0.8615	1.0769	0.0042	207.86	219.76	219.79	1.06
Cotri-mox 480 mg tabs	0.0154	0.0154	0.0308	0.0116	1.29	2.67	1.33	2.07
Diazepam 5 mg tab/cap	0.0139	0.0062	0.0366	0.0189	0.63	2.12	0.73	3.33
Diclofenac 50 mg tab/cap	0.0154	0.0079	0.0154	0.0127	0.55	1.18	1.21	2.14
Doxycycline 100 mg cap	0.0308	0.0185	0.0308	0.0192	0.99	1.61	1.60	1.63
Fefol 200 mg + 0.5 mg cap	0.0462	0.0308	0.0615	0.0314	0.89	1.75	1.47	1.96
Fluconazole 200 mg tab	0.0354	0.0308	0.0939	0.0698	0.53	2.42	0.51	4.57
Fluoxetine 20 mg cap	0.0769	0.0369	0.1692	0.0103	3.11	9.22	7.47	2.97
Furosemide 40 mg tab	0.0308	0.0208	0.0308	0.0062	3.23	5.00	4.97	1.55
Glibenclamide 5 mg tab/cap	0.0185	0.0092	0.0308	0.0053	1.70	4.15	3.49	2.44
Haloperidol 5 (0.5) mg tab	0.0308	0.0205	0.0477	0.0572	0.40	1.08	0.54	2.70
Hydrocortisone (50 mg/ml) inj	1.3846	0.7692	2.1538	0.520	0.95	3.55	2.66	3.75
Hyoscine BB 10 mg tab	0.1077	0.0923	0.1231	0.0421	2.49	2.64	2.56	1.06
Ibuprofen 400 mg tab	0.0308	0.0308	0.0308	0.0132	2.35	2.35	2.33	1.00
Loperamide 2 mg cap	0.2923	0.0923	0.4615	0.0103	–	28.35	28.38	–
Metformin 500 mg tab	0.0308	0.0215	0.0392	0.0162	1.36	1.91	1.90	1.41
Metoclopramide HCl 10 mg tab	0.0154	0.0092	0.0308	0.0081	1.11	3.83	1.90	3.44
Metronidazole 250 mg caps	0.0154	0.0154	0.0308	0.0067	2.24	2.54	2.30	1.13
Omeprazole 20 mg tab/cap	0.0215	0.0171	0.0308	0.0154	1.04	2.01	1.40	1.94
ORS sack (1 L)	0.2154	0.0923	0.3077	0.0561	1.43	5.49	3.84	3.85
Paracetamol 24 mg/ml susp	0.9231	0.6308	0.9231	0.0064	72.19	144.22	144.23	2.00
Paracetamol 500 mg tab	0.0154	0.0069	0.0185	0.0058	1.03	2.59	2.66	2.50
PTU 50 mg tab	0.1446	0.1139	0.2692	0.0718	2.02	3.75	2.01	1.86
Salbutamol 0.1 mg/dose	3.0769	2.0615	3.6923	0.0058	355.52	583.62	530.50	1.64
Risperidone 1 mg tab	0.2154	0.0462	0.2246	0.0375	5.55	7.47	5.74	1.35
Simvastatin 20 mg tab/cap	0.2062	0.0862	0.2357	0.0163	–	12.64	12.65	–
TTC 1% ointment	0.3077	0.1846	0.3077	0.1294	1.43	2.38	2.38	1.66
Valproate 200 mg tab	0.0923	0.0735	0.1308	0.1755	0.44	0.70	0.53	1.58

### Affordability of essential medicines

Majority of the medicines were found to be unaffordable, costing more than one day wage in both private and public facilities. The percentage of unaffordable medicine were 72.09 and 91.84 for public and private facilities, respectively, with 79.17% of the medicines were unaffordable when both settings are combined. The result of the overall affordability calculation revealed that ceftazidime, risperidone, and ampicillin injection were the top three unaffordable medications requiring 171.33, 73.43 and 58.74 days wage of the lowest paid government employee, respectively. The top three unaffordable medications in the private facilities were ceftazidime, risperidone and valproate requiring 186.01, 95.45 and 62.89 days wage of the lowest paid government employee, respectively. While in the public facilities risperidone takes the lead with 70.91 days wage followed by ceftazidime and valproate with 53.84- and 39.88-days wage, respectively (Table 6).

**Table 6:** Affordability of essential medicines in eastern Ethiopia

Generic versions	DDD	# Units	# Days	Total no. of dose	Public	Public MP	Public afford	Private	Private MP	Private afford	Overall	Overall MP	Overall afford
Adrenaline 1 mg/ml inj	NA	1 vial	7	7	….	…	….	0.138	0.966	2.195	0.1385	0.9695	2.203
Albendazole 200 mg tab	0.4 g	2 tab	3	6	0.031	0.186	0.423	0.077	0.462	1.05	0.0615	0.369	0.839
Allopurinol 100 mg tab	0.4 g	4 tab	30	120	0.032	3.84	8.727	0.06	7.2	16.364	0.0338	4.056	9.218
Amiodarone 200 mg tab	0.2 g	1 tab	30	30	…	…	…	…	…	…	…	…	…
Amitriptyline 25 mg tab/cap	75 mg	3 tab	30	90	0.019	1.71	3.886	0.054	4.86	11.045	0.0462	4.158	9.45
Amox 250 mg cap	1.5 g	6 cap	7	42	0.015	0.63	1.432	0.031	1.302	2.959	0.0246	1.0332	2.348
Amox 500 mg cap/tab	1.5 g	3 cap	7	21	0.031	0.651	1.479	0.046	0.966	2.195	0.0369	0.7749	1.761
Ampicillin 500 mg inj	6 g	12 vial	7	84	0.154	12.936	29.4	0.462	38.808	88.2	0.3077	25.8468	58.742
ASA 100 mg tab	1 tablet	1 tab	30	30	…	…	…	0.092	2.76	6.272	0.0923	2.769	6.293
ASA 300 mg tab	3 g	10 tab	7	70	0.015	1.05	2.386	0.015	1.05	2.386	0.0154	1.078	2.45
Atenolol 50 mg tab/cap	75 mg	1.5 tab	30	45	0.023	1.035	2.350	0.031	1.395	3.170	0.0308	1.386	3.15
Azithromycin 250 mg cap	0.3 g	1 cap	7	7	0.123	0.861	1.957	0.462	3.234	7.35	0.3591	2.5137	5.713
Beza Pen G 2.4 MIU	6 MIU	7 vial	7	49	0.28	13.72	31.181	0.462	22.638	51.45	0.3077	15.0773	34.267
Bisacodyl 5 mg tab	10 mg	2 tab	7	14	0.023	0.322	0.732	0.062	0.868	1.973	0.0308	0.4312	0.98
Bisoprolol 5 mg/tab	10 mg	2 tab	30	60	…	…	…	0.077	4.62	10.5	–	…	…
Captopril 25 mg tab	50 mg	2 tab	30	60	0.031	1.86	4.227	0.046	2.76	6.273	0.0308	1.848	4.2
Carbamazepine 200 mg tab	1 g	5 tab	30	150	–	…	…	0.088	13.2	30	0.0885	13.275	30.170
Ceftazidime 1 g inj	4 g	4 vial	7	28	0.846	23.688	53.836	2.923	81.844	186.009	2.6923	75.3844	171.328
Ceftriaxone 1 g vial	2 g	2 vial	7	14	0.615	8.61	19.568	0.769	10.766	24.468	0.6462	9.0468	20.561
Cipro 500 mg tab/cap	1 g	2 tab	7	14	0.031	0.434	0.986	0.062	0.868	1.973	0.0615	0.861	1.957
Clindamycin 150 mg	1.2 g	8 cap	7	56	0.129	7.224	16.418	0.097	5.432	12.345	0.1151	6.4456	14.649
Cloxa 500 mg cap/tab	2 g	4 cap	7	28	0.045	1.26	2.864	0.062	1.736	3.945	0.0462	1.2936	2.94
Cotri-mox 240/5 susp		1 bottle	7	1	0.873	0.873	1.984	0.923	0.923	2.098	0.9231	0.9231	2.098
Cotri-mox 480 mg tabs	4 tab	4 tab	7	28	0.015	0.42	0.955	0.031	0.868	1.973	0.0154	0.4312	0.98
Diazepam 5 mg tab/cap	10 mg	2 tab	7	14	0.012	0.168	0.382	0.04	0.56	1.273	0.0139	0.1946	0.442
Diclofenac 50 mg tab/cap	0.1 g	2 tab	7	14	0.007	0.098	0.223	0.015	0.21	0.477	0.0154	0.2156	0.49
Doxycycline 100 mg cap	0.1 g	1 tab	7	7	0.019	0.133	0.302	0.031	0.217	0.493	0.0308	0.2156	0.49
Fefol 200 mg + 0.5 mg cap	1 cap	1 cap	30	30	0.028	0.84	1.909	0.055	1.65	3.75	0.0462	1.386	3.15
Fluconazole 200 mg tab	0.2 g	1 tab	7	7	0.037	0.259	0.589	0.169	1.183	2.689	0.0354	0.2478	0.563
Fluoxetine 20 mg cap	20 mg	1 cap	30	30	0.032	0.96	2.182	0.095	2.85	6.477	0.0769	2.307	5.243
Furosemide 40 mg tab	40 mg	1 tab	30	30	0.02	0.6	1.363	0.031	0.93	2.114	0.0308	0.924	2.1
Glibenclamide 5 mg tab/cap	10 mg	2 tab	30	60	0.009	0.54	1.227	0.022	1.32	3	0.0185	1.11	2.523
Haloperidol 5 (0.5) mg tab	8 mg ?	2 tab	30	30	0.023	0.69	1.568	0.062	1.86	4.227	0.0308	0.924	2.1
Hydrocortisone (50 mg/ml) inj	30 mg	–	7	7	0.492	3.444	7.827	1.846	12.922	29.368	1.3846	9.6922	22.027
Hyoscine BB 10 mg tab	1 tab	1 tab	7	7	0.105	0.735	1.670	0.111	0.777	1.766	0.1077	0.7539	1.713
Ibuprofen 400 mg tab	1.2 g	3 tab	7	21	0.031	0.651	1.479	0.031	0.651	1.479	0.0308	0.6468	1.47
Loperamide 2 mg cap	10 mg	5 cap	7	35	…	…	…	0.292	10.22	23.227	0.2923	10.2305	23.251
Metformin 500 mg tab	2 g	4 tab	30	120	0.022	2.64	6	0.031	3.72	8.455	0.0308	3.696	8.4
Metoclopramide HCl 10 mg tab	30 mg	3 tab	7	21	0.009	0.189	0.429	0.031	0.651	1.479	0.0154	0.3234	0.735
Metronidazole 250 mg caps	2 g	8 cap	7	56	0.015	0.84	1.909	0.017	0.952	2.164	0.0154	0.8624	1.96
Omeprazole 20 mg tab/cap	20 mg	1 cap	7	7	0.016	0.112	0.254	0.031	0.217	0.493	0.0215	0.1505	0.342
ORS sack (1 L)		1 sac	7	7	0.08	0.56	1.272	0.308	2.156	4.9	0.2154	1.5078	3.427
Paracetamol 24 mg/ml susp		1 bottle	7	1	0.462	0.462	1.05	0.923	0.923	2.098	0.9231	0.9231	2.098
Paracetamol 500 mg tab	3 g	6 tab	7	42	0.006	0.252	0.572	0.015	0.63	1.432	0.0154	0.6468	1.47
PTU 50 mg tab	0.1 g	2 tab	30	60	0.145	8.7	19.772	0.269	16.14	36.681	0.1446	8.676	19.718
Salbutamol 0.1 mg/dose	0.8 mg	8 d/day	30	1	2.062	2.062	4.686	3.385	3.385	7.693	3.0769	3.0769	6.993
Risperidone 1 mg tab	5 mg	5 tab	30	150	0.208	31.2	70.909	0.28	42	95.454	0.2154	32.31	73.432
Simvastatin 20 mg tab/cap	30 mg	1.5 tab	30	45	…	…	…	0.206	9.27	21.068	0.2062	9.279	21.089
TTC 1% ointment	1 tube	1 tube	7	1	0.185	0.185	0.420	0.308	0.308	0.7	0.3077	0.3077	0.699
Valproate 200 mg tab	1.5 g	7.5 tab	30	225	0.078	17.55	39.886	0.123	27.675	62.898	0.0923	20.7675	47.199

*DDD* defined daily dose, *MP* median price, *afford affordability*

## Discussion

Access to essential medicines is a universal human right and availability and affordability are the preconditions for it [21, 22]. In line with the sustainable development goals, WHO has outlined a framework that assists the policy makers to improve access to essential medicine for universal health coverage by 2030. The four major components of access are rational selection and use of medicines, availability and affordability, sustainable healthcare financing, and reliable supply system of quality products [22–24]. In this regard, essential medicines should be systematically selected using evidence-based approach with due consideration on public health priority, comparative cost-effectiveness, efficacy, safety, and generic versions, among others. The provision of complete healthcare is realized when essential medicines are available in the required quality, quantity, and at all times and in a way that patients can easily afford [22, 23, 25].

However, the availability of essential medicines is still suboptimal in several low-income countries. In particular, the availability of pediatric formulations and key medicines for chronic diseases is still suboptimal even in middle-income countries [26]. As per the global action plan of WHO, the proposed 80% target for access to essential medicines is the key to attain the overall target of 25% relative reduction in premature mortality from chronic non-communicable diseases (NCDs) by 2025 [27]. Besides, improving the availability and affordability of essential medicines is likely to enhance their use and help towards in achieving WHO targets of 50% use of key medicines by 2025 [28].

In this regard, this study has addressed the availability, price and affordability of 50 essential medicines in public and private health facilities of eastern Ethiopia. Generally, nearly half of OB medicines, 42.85% OBs from WHO/HAI core drugs, were totally absent in all health facilities included in the survey. The overall availability of OB medicines was lower in public facilities. Besides, nearly half (52%) of surveyed essential medicines were available in only 50% or more of the facilities studied. Only eight LPG versions (16.0%) were available in 80% or so of the facilities surveyed. The overall availability of LPG versions was higher in private drug retail outlets. Except ceftriaxone and hydrocortisone, all imported LPG versions were available in less than 50% of the facilities included. Chemotherapeutic agents were the most commonly available class in both public and private settings. The availability index for drugs for chronic diseases was lower than that used for acute conditions.

The median buyers’ prices for 94% LPG versions were significantly higher in private drug retail outlets. Moreover, the private median price of LPGs were more than three times that of the public sector for 16% of drugs. The MPR value indicated that median price of LPGs in the private sector was more than four times the IRP in 30% of drugs. In public sector, about 16% of LPGs had a median price of more than three times than that of IRP. With reference to the lowest paid government employee, majority of LPG medicines were found unaffordable, costing more than one day wage in both public and private facilities. Generally, four out of five essential medicines were found unaffordable in Ethiopian healthcare settings with the worst price escalation being observed in private settings (nine drugs out of ten essential medicines). In low-income countries like Ethiopia, low availability with high buyers’ price and low affordability vividly reflects a failure of implementing national drug policy on essential medicines.

Unlike this study, the availability of OBs exceeded the WHO target of 80% and found affordable in Qatar public health facilities, although 30% of surveyed medicines were beyond the acceptable threshold of 4.0 in private sector [29]. Compared with this study, study conducted in the northern Ethiopia indicated that there was lower overall availability (34.1%) but better affordability of LPGs 30% and 50% of LPGs demanded more than a single daily wages to purchase these drugs in public and private sectors, respectively [30]. In Jordan, much better availability of LPGs was observed in both public (72%) and private (76%) sectors for chronic diseases and the prices of medicines in public sectors were generally affordable but not in private settings [31]. Likewise, in upper-middle income countries like Malaysia, the affordability of all generic versions of essential medicines was below 2-day wages of the lowest paid government employees in the public sector [32].

In our study, eight drugs (16.0%) met the WHO target of 80%. It was in trajectory with the study in which 15.2% and 18.9% of LPGs met WHO target in the public and private sectors of low-income countries, respectively. This value was 7 to 8% higher in lower-middle income countries [33]. Besides, a study conducted in Tanzania and central Ethiopia indicated that locally produced products had greater mean availability (48%) than that of imported ones (19%) [34] indicating the need of more local manufacturing plants for better access of essential medicines.

In a study conducted on six low-and middle-income countries, less than 10% surveyed medicines were available in public sector in four of the countries surveyed [35]. Unlike high-income countries, low- and middle-income countries usually have poor regulation of pharmaceutical markets and often lack feasible purchasing and pricing strategies [36]. Country specific studies indicated that better availability and more affordable generic versions were reported from Rwanda [37] and Nepal [38]. Relatively higher availability of OBs in both public (6.8%) and private (55.0%) facilities were also observed in Pakistan whereas the availability of generic versions was lower in public (35.3%) and private (20.3%) facilities [39]. In a study conducted in China, higher availability of pediatric OBs were observed in public (7.5%) and private (8.9%) sectors although the overall availability of generic versions in both public (34.2%) and private (29.4%) sectors were by far lower but more affordable compared to our study [40]. What is more, in the primary care settings of Vietnam, the availability of essential medicines was higher (56.4%) than our study. Likewise, the study conducted in eleven countries of the Asian Pacific region demonstrated that there was slightly higher availability (56.7%) of generic versions of essential medicine in the private sector though it was found lower (35.5%) in the public sector [41]. In the upper middle-income and high income-countries, the availability of OBs and LPGs was by far higher in both public and private sectors with less price variation and more affordability compared to our study [42–45]

Regarding chronic diseases in particular, a significant proportion of patients in low- and middle-income countries do not have access (low availability and/or low affordability) to generic versions of essential medicines for the treatment of hypertension [46], diabetes [47–49], chronic respiratory diseases including bronchial asthma [27], diabetes and hypertension combined [50], several non-specific NCDs [51–53]. Multilevel analysis also indicated that the availability and affordability of essential diabetes medicines were significantly associated with their use [47]. Likewise, a study conducted in China indicated that high cost medications were more likely to be prescribed than lower cost alternatives and only one-third of facilities stocked high value (essential) medicines [54]. To this end, medicines take a large proportion of household expenditure on health in low-and middle-income countries. According to WHO survey, up to 9.5% of the total expenditure was spent on medicines and is almost three times higher than the one spent in high-income countries [21, 22, 55]. Inadequate healthcare financing and inefficient and unreliable supply system is attributable to high out-of-pocket expenditure in such resource poor settings. The PURE study also indicated that secondary prevention medicines for cardiovascular diseases were found unavailable and unaffordable in large proportion of customers in low- and middle-income countries [28].

With regard to AWaRe (Access, Watch and Reserve) classification of antibiotics, there has been a declining trend of at least 60% total consumption of antibiotics (WHO-national level target) in the access category from 76% in 2000 to 55% in 2015. Without policy intervention affecting the availability of such essential antibiotics, it is difficult to attain at least 60% consumption of antibiotics from ‘Access’ category by 2023 [56]. In our study, all the included essential antibiotics were from both Access and Watch categories with the former accounting nearly two-thirds of the total agents.

Even in countries where there is drug pricing information, the availability of medicines in public sectors is about one-third while that of the private sector is about two-thirds, and the buyers’ prices for LPGs vary from 2.5 to 6.5 MPRs in these two sectors [21]. In this regard, a multitude of strategies including managerial, regulatory, economic, and educational approaches shall be devised to increase the access of essential medicines in the public sector [21, 35, 41, 57]. Economic strategies including competitive or pooled procurement policies for multisource products, price negotiation for sole source products, reducing taxes and tariffs and regulating mark-ups, provision of community-based health insurance, and sustainable health care financing shall be taken as the prior agenda for Ethiopia to address all segments of the population. In addition, regulation of the pharmaceutical market, strict implementation of generic procurement policies, efficient and evidence-based procurement, provision of vivid pricing and procurement information, as well as installation of local manufacturing plants shall also be considered to increase the access of essential medicines.

### Strength and limitations

Using validated WHO/HAI methodology allows for the measurement of medicine availability and prices in a reliable and standardized way. Utilization of international reference prices can also allow for valid international comparisons between Ethiopia and other countries. Besides, we considered global core, regional and national essential medicines for international comparison. However, being a single point cross-sectional study, it is unable to reflect the average monthly or annually availability of medicines at individual outlets. The affordability section is also heavily dependent on the economic status, public salary scale, and exchange power of Ethiopian birr and subject to change over time.

## Conclusion

The overall availability of generic versions of essential medicines was by far lower than the WHO target of 80% with 16% of the surveyed medicines surpassing the cut-off point. The overall availability of OBs was also less than 5%. About 30% of drugs in the private sector had a price of more than four times (MPR threshold) than that of the international references. Moreover, four out of five drugs were found unaffordable when both settings were combined. Looking at the private sector, about nine from ten drugs demanded several days of wages of lowest paid government employees. There is a higher tendency of prescribing generics than the OB versions of essential medicines as the OB versions are much more expensive in such resource limited settings. However, much is yet to be invested in controlling the price of drugs. Ensuring access of essential medicines is one of the general objectives of Ethiopian National Drug Policy. In this regard, the current regional study indicates the availability and affordability is suboptimal which calls the responsible stakeholders to devise a strategy that help increase the access of essential medicines and rescue the struggling healthcare system.

## Data Availability

All the data used for the study are contained within the manuscript.
